# Comprehensive Resilience Assessment of Global Staple Food Trade Networks Based on Structural Evolution and Cascading Failures

**DOI:** 10.3390/foods15122169

**Published:** 2026-06-16

**Authors:** Shu Zhou, Lei He

**Affiliations:** School of Economics and Management, Shaanxi University of Science and Technology, Xi’an 710021, China; 250711003@sust.edu.cn

**Keywords:** food trade network, underload cascading failure, interdependent network, vulnerability

## Abstract

Amid intensifying extreme climate events, geopolitical conflicts, and sudden trade policy disruptions, the resilience and vulnerability of global staple food trade systems have emerged as pressing governance concerns. This study constructs directed weighted trade networks for wheat, maize, and rice from 2015 to 2024 and evaluates their vulnerability and resilience evolution using a three-dimensional structural resilience framework and underload cascading failure models. The results reveal that all three networks display scale-free and disassortative properties. The wheat network gradually recovered following the Russia–Ukraine conflict, whereas structural imbalance continues to deepen in the maize network, and the rice network faces persistent resilience pressure arising from excessive dependence on core exporters. Cascading failure simulations indicate that targeted attacks on key exporting countries can trigger large-scale network collapse. Introducing cross-crop substitution effects markedly enhances the resilience of individual food trade networks through cross-layer substitution and supplementation; yet under simultaneous attacks, crop substitution effects instead serve as a conduit for cross-layer cascading failure propagation, and even a minimal willingness to substitute can weaken network resilience. Accordingly, this study proposes policy recommendations to strengthen the resilience of the global staple food trade network.

## 1. Introduction

As global reliance on international trade to meet regional nutritional needs intensifies, the distribution of agricultural resources has accelerated dramatically over the past two decades [1], with global trade volumes surging by 133% and far outpacing the growth in actual food production. Amid this rapid growth, four core crops, namely soybeans, wheat, maize, and rice, have played a dominant role, accounting for approximately 28% of the global food supply gap and underpinning more than 60% of global staple food consumption [2,3]. Within such an immense supply–demand matching system, the grain trade network serves as an indispensable foundational infrastructure, providing a crucial buffer against localized food shortages and ensuring the smooth operation of supply chains [4,5]. Yet as the volume and frequency of network transactions have grown increasingly complex, the inherent vulnerabilities of this highly interconnected system have become ever more apparent, emerging as a central issue of global concern [6,7]. The stability of global trade networks remains under persistent threat from multiple environmental and human-induced disruptions, ranging from extreme weather events such as severe droughts and floods to abrupt export bans, often triggering sharp food price spikes and exacerbating global food insecurity [8,9]. Even more concerning, the COVID-19 pandemic and the Russia–Ukraine conflict, along with other disruptive macroeconomic shocks, have placed severe strain on the safety net of the global food system in recent years [10,11]. In particular, the Russia–Ukraine conflict has severely disrupted global wheat supply chains, prompting UN reports of record-high global food prices in March 2022 and directly driving food price spirals in European countries such as Germany, Italy, France, and Spain [12,13,14]. Therefore, in-depth research into the mechanisms underlying food trade network vulnerabilities, aimed at assisting governments in developing strategies to enhance supply chain resilience against long-term crises, has become an urgent need. Existing research has approached food security and trade resilience from multiple dimensions. Analyses of multi-commodity trade networks reveal that network expansion does not yield evenly distributed resilience, and systemic vulnerability becomes especially pronounced when core nodes suffer deliberate attacks. PageRank [15] studies from a virtual water trade perspective have incorporated water resource constraints into global agricultural systems, thereby improving assessment methods [16]. The nutritional effects of agricultural trade have also drawn attention, with evidence confirming that the globalization of food systems reshapes nutritional linkages across countries of different income levels [17]. From an institutional perspective, ecological conservation redline policies are regarded as providing critical support for food production capacity and offer valuable insights for food security governance in developing countries [18]. Empirical research based on U.S. data uncovers the complex dynamics among agricultural value added, reservoir management, and food security, highlighting the essential roles of innovation and water resource efficiency [19]. The interactions between climate change and food security have likewise been systematically reviewed, and resilience-enhancing pathways such as cross-sectoral collaboration and sustainable production have been successively proposed [20].

Scholars have employed a range of analytical approaches to accurately capture the complex flows of agricultural inputs at national and global scales [12,13,14]. Early macroeconomic studies relied heavily on traditional gravity models, which effectively captured the growing interconnectedness of global food markets [21]. However, trade networks consist of numerous nodes and intricate interconnections, restricting conventional gravity models from depicting fine-grained topological characteristics and multi-dimensional structural properties [22,23]. Network analysis models were introduced and developed to overcome this methodological limitation, with early applications effectively unraveling the inherent structural complexity of wheat trade [24]. Subsequent studies have further refined this framework, extending beyond static structure characterization to incorporate the assessment of network resilience to food supply shocks [25]. Subsequently, this analytical framework has been extensively applied to other food commodities, revealing the potential cascading effects of trade disruptions and the inherent vulnerabilities of the overall trade network [12,13,14]. While existing network research has provided valuable insights into the structural and dynamic evolution of trade networks [12,13,14], the current literature remains limited in that most studies examine trade flows of individual grains in isolation. This study introduces the concepts of network resilience and coupled cascading failure models from complex network theory to elucidate the collapse mechanisms of major food trade systems under extreme external shocks, thereby establishing a rigorous methodological foundation for tracing how localized trade disruptions escalate into global paralysis. However, existing research still lacks a unified theoretical framework to explain how localized trade disruptions escalate into systemic crises through multi-commodity and multi-tiered coupling channels. Static or dynamic assessments of a single commodity cannot adequately capture the cross-commodity substitutions and cross-tier feedback effects inherent in real-world food systems [26].

With the adoption of the complex network paradigm, network resilience has emerged as a central framework for analyzing the capacity of trade systems to withstand extreme macroeconomic shocks. Originating in ecology as a measure of a system’s ability to absorb external disturbances [27], the concept was subsequently widely adopted in complex systems science. Within network theory, network resilience is defined as a systemic property whereby a network adaptively modifies behavior upon node or edge damage, thereby averting an irreversible tipping point, maintaining critical topological functions, and achieving state recovery [28]. Early quantitative resilience research relied primarily on static topological metrics, employing intrinsic structural properties such as effective resistance centrality to assess overall network robustness and identify vulnerable hubs [29], while Table 1 presents the multiple metrics currently used to evaluate static toughness. Yet static characteristics cannot fully capture the dynamic stress responses observed in the real world; consequently, resilience modeling grounded in system dynamics has gradually become the mainstream paradigm [30] and has been widely applied to commodity trade networks, which exhibit strong structural similarities. For example, recent research has quantified the risk absorption capacity of specific supply chains, such as the cobalt supply chain, against external shocks by constructing a quantitative framework that incorporates clustering and hierarchy and by simulating deliberate sabotage and random disruptions [31]; and by tracking how network performance degrades under intensifying deliberate attacks, the study precisely uncovers the inherent vulnerabilities of the trade system [31]. These simulation studies, focused on node removal and performance degradation, establish a solid foundation for characterizing both the inherent robustness and the adaptive capacity a network displays against initial shocks. Yet real-world crises in global trade systems are often more complex: the failure of a local core node rarely remains isolated but tends to transcend a single node, triggering deeper and more destructive crises along trade links. Such crises, triggered by local disturbances, are termed cascading failures in complex network theory. While early trade resilience research rarely addressed specific risk transmission mechanisms, classical theories from network dynamics offer powerful analytical tools to fill this gap. Existing mainstream cascade models, such as the load-capacity model, the sandpile model, CASCADE, and the OPA model, all revolve around three fundamental elements: the initial load of nodes, their maximum capacity, and the rules governing load redistribution following failure [32,33,34]. Specifically, the definition of initial load has evolved from a simple uniform distribution, through median centrality–based models, to currently prevalent complex functional forms that incorporate both the degree of a node and that of its neighbors [35]. Meanwhile, node capacity settings typically fall into three categories: those independent of the initial load, capacity settings strictly proportional to the initial load, and those governed by a nonlinear mapping [36,37,38]. When a node is overloaded to failure by an extreme shock, excess load redistribution serves as the last line of defense against crisis propagation. Networks formed by coupling nodes of different degrees exhibit substantially greater robustness than those with equal-degree topologies [39]. Various allocation mechanisms have been extensively investigated, including equal distribution, random walks, global coordination, and local optimization, among which dynamic reallocation strategies with adjustable parameters offer a novel approach to enhancing network resilience and suppressing cascading propagation [35,40]. Although existing network studies have provided valuable insights into the structure and dynamic evolution of trade networks, the current literature has notable limitations. First, in terms of research focus, most studies examine trade flows of a single grain in isolation [41], overlooking the cross-level risk transmission arising from consumer-end substitutability among wheat, corn, and rice. Second, methodologically, traditional structural indicators assess resilience from a static topological perspective and single-level coupling models from a dynamic diffusion perspective, thus lacking a multidimensional, three-dimensional evolutionary analysis and dynamic simulation-based vulnerability assessments from the standpoint of coupled grain trade [42,43]. To substantively reveal the collapse mechanisms of the major grain trade system under extreme external shocks, we introduce a three-dimensional structural resilience framework coupled with a cascade failure model, thereby providing a rigorous methodological foundation for investigating how localized trade disruptions escalate into systemic collapse.

Given the three gaps outlined above, the theoretical contribution of this study lies in constructing a unified analytical framework composed of three progressive layers: three-dimensional structural evolution, underload cascades, and cross-commodity coupling. This framework advances the current understanding of resilience in global staple food trade networks. Specifically, to address the limitation that static topological indicators fail to capture dynamic stress-response processes, this study constructs directed weighted trade networks for the three major staple grains, namely wheat, corn, and rice, using UN Comtrade data from 2015 to 2024. This period encompasses a range of systemic shocks, including the COVID-19 pandemic, the Russia–Ukraine conflict, extreme weather events, and export bans imposed by multiple countries, thereby providing a representative observation window for examining the evolution of trade network resilience. Concurrently, a three-dimensional dynamic evolution framework is introduced, shifting resilience measurement from a static, snapshot-based assessment to a dynamic, trajectory-based assessment and enabling the characterization of structural recovery pathways under shocks such as the Russia–Ukraine conflict [61,62]. Second, we embed an underloaded cascade failure model into this dynamic framework and design four targeted attack strategies. Through multi-perspective simulations of attacks on the trade network over the past decade, including those based on PageRank and median centrality, we overcome a key limitation of existing node-removal studies that assess only the risk absorption capacity of a single commodity [13]. Most crucially, this study introduces multi-layer coupled networks along with adjustable substitution willingness parameters to endogenize cross-commodity substitution effects at the consumer level within the cascade process. This reveals a counterintuitive mechanism overlooked by previous single-layer models [63]: under synchronized attacks, substitution effects actually serve as a transmission channel for cross-level cascade failures, and even a marginal willingness to substitute can weaken overall resilience. These findings provide new theoretical evidence for the policy debate in food security governance on whether promoting crop diversification necessarily enhances resilience.

## 2. Data Sources and Research Methodology

### 2.1. Data Description and Network Construction

Based on the FAO Food Security Statistics Framework, international food trade statistical conventions, and the classification standards of the WTO Agreement on Agricultural Products, wheat, maize, and rice serve as core crops for global staple food consumption and strategic reserves, and their trade flows directly reflect the fundamental landscape of food security. Accordingly, this study draws upon wheat, maize, and rice trade data (HS codes 1001, 1005, and 1006) from the UN Comtrade database covering 2015–2024 to construct the respective food trade networks. To maintain data consistency and adhere to the World Bank technical paper (Imports, Exports and Mirror Data with UN COMTRADE (World Bank, 2024)), this study relies on import statistics. Trade volumes are analyzed primarily by weight rather than monetary value to avoid biases introduced by price fluctuations.

The selection of the 2015–2024 analysis interval is motivated by two primary considerations. First, this time span fully encompasses a series of typical events that have recently generated systemic disruptions to the global food trade system, including the agricultural tariff disputes during the China–US trade friction of 2018–2019, the supply chain shocks triggered by the COVID-19 pandemic from 2020 onward, production shortfalls in major exporting countries due to extreme weather in 2021–2022, the wheat and fertilizer supply crisis caused by the Russia–Ukraine conflict in 2022, and India’s rice export ban in 2023 [10,13,14]. Meanwhile, in terms of policy and statistical baseline comparability, the year 2015 marks the starting point at which food trade statistics among major economies stabilized under the HS 2012 revision, thereby ensuring longitudinal comparability of trade data across the decade.

Based on complex network theory, this study constructs three types of directed weighted trade networks $G_{1}=(N,E,W_{1})$ for wheat, maize, and rice covering the period 2015–2024, where $N=\left\{n_{i}|i=1,2,\ldots \}\right.$ denotes the set of countries (or regions) participating in the trade of these three staple grains, E denotes the matrix of directed supply relationships among countries (or regions), and $W_{1}$ denotes the weight matrix of the directed supply relationships. Specifically, if country (or region) $n_{i}$ imports a certain staple grain from or exports it to country (or region) $n_{j}$, then $e_{\mathrm{ij}}$ or $e_{ji}=1$, with the corresponding weight equal to $w_{ij}$ or $w_{ji}$; otherwise, $e_{\mathrm{ij}}$ or $e_{ji}=0$, and $w_{ij}$ or $w_{ji}=0$.

$$E=\left[\begin{array}{cccc} 0 & e_{12} & \ldots & e_{1j}\\ e_{21} & \ldots & \ldots & \ldots \\ \vdots & \ldots & \ldots & e_{i-1,j}\\ e_{i1} & \ldots & e_{i,j-1} & 0 \end{array}\right],\;W_{1}=\left[\begin{array}{cccc} 0 & w_{12} & \ldots & w_{1j}\\ w_{21} & \ldots & \ldots & \ldots \\ \vdots & \ldots & \ldots & w_{i-1,j}\\ w_{i1} & \ldots & w_{i,j-1} & 0 \end{array}\right]$$

### 2.2. Research Framework

**Figure 1:**
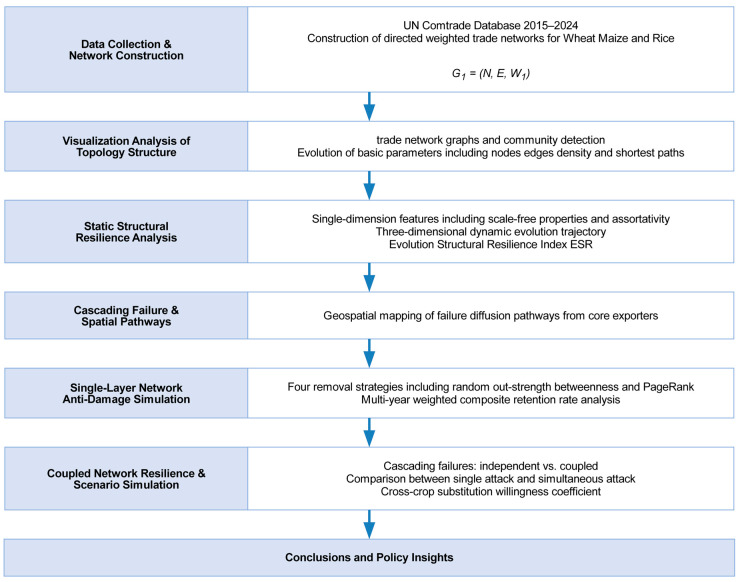
Research framework flowchart.

Evaluating trade network resilience is inherently multidimensional. A global lens examines overall structural stability and resource transfer efficiency, whereas a local perspective captures how critical nodes absorb shocks and recover from external disruptions. This study follows a progressive approach, advancing from static topological characterization through dynamic evolution tracking to resilience stress testing, to comprehensively characterize the complex features of the global staple food trade network and construct a systematic and comprehensive analytical framework, as illustrated in Figure 1.

For static resilience assessment, this study constructed directed weighted trade network models for wheat, maize, and rice based on complex network theory, and analyzed the structural characteristics and evolution of these three major staple grain trade networks from 2015 to 2024. Furthermore, the study introduced a three-dimensional dynamic evolution framework to assess the static structural resilience of these networks from both one-dimensional and three-dimensional perspectives.

For dynamic resilience assessment, this study employs an underload cascade failure model to simulate the adaptive capacity of staple food networks under disruptive disturbances. The evaluation proceeds across three dimensions, progressing from the most basic to the most complex: first, tracking the sequential propagation pathways across the global landscape following the failure of key exporting countries; second, performing single-layer network simulations to assess the vulnerability of individual staple foods to specific attack patterns via random disturbances and various targeted node removal strategies; and third, this study constructs a multi-layered coupled network to simulate individual-network attacks and synchronized attacks across multiple networks, and to assess the variation in resilience retention under different substitution-willingness scenarios.

### 2.3. Structural Resilience Based on Three-Dimensional Space Evolution

Network topology provides a foundational framework for analyzing complex systems by viewing a system as a structure composed of nodes and edges, revealing how components interact and integrate into a whole [46]. Subsequently, graph theory methods can investigate the system, uncovering organizational information through the characterization of topological and spectral properties and key components. Newman (2003) argues that topological structure is central to understanding complex systems [64], whereas Crespo et al. (2014) developed a regional resilience evolution framework that centers on the structural characteristics of local-level knowledge networks, and suggested that degree distribution and degree correlation serve to explain how clusters combine technological lock-in with regional lock-out, proposing a simple statistical indicator for measuring cluster structure [65].

Existing static network resilience measures predominantly rely on unidimensional structural attributes, such as connectivity efficiency, betweenness centrality, and degree correlation, as summarized in Table 1. A common limitation of these indicators is their single-dimensional nature, capturing only the scalar magnitude of resilience change rather than its directional structure. Moreover, they provide static snapshots that cannot trace spatial evolutionary trajectories and offer limited explanatory power for the causes of resilience changes. When resilience declines, it remains unclear whether the decline stems from redundancy loss, core concentration, or assortativity deterioration. To address these limitations, this study introduces the three-dimensional structural resilience framework proposed by Yu, Ma, and Qian (2023b) [31] and further integrates clustering, hierarchy, and matching analyses to evaluate the structural resilience of wheat, corn, and rice trade networks.

The x-axis, representing the average clustering coefficient [53] defined in Equation (1), captures the variation in clustering strength across the staple food trade networks and thereby measures the degree of concentration in staple food trade among individual countries (or regions).

(1) $$C_{wi}=\left(\sum_{i=1}\frac{{\left({K_{i}}^{in}+{K_{i}}^{out}\right)}_{ii}^{3}}{2d_{i}^{tot}\left(d_{i}^{tot}-1\right)-2d_{i}^{↔}}\right)/len\left(N\right)$$

where ${K_{i}}^{in}$ and ${K_{i}}^{out}$ denote the weighted in-strength and out-strength of the node; $d_{i}^{tot}$ represents the sum of the in-degree and out-degree of the node; $d_{i}^{↔}$ indicates the number of nodes that have bidirectional edges with the node and len(N) denotes the total number of nodes in the network.

The Y-axis, representing the node strength distribution coefficient [55] defined in Equation (2), captures the variation in hierarchical strength across the wheat, maize, and rice trade networks and thereby measures the degree of concentration in the distribution of countries (or regions) within major grain trade.

(2) $$K_{i}^{tot}=C{\left(K_{i}^{*tot}\right)}^{\alpha }LnK_{i}^{tot}=LnC+\alpha LnK_{i}^{tot}$$

where $K_{i}^{tot}$ denotes the total strength, defined as the sum of in-strength and out-strength of the node, C is a constant, $K_{i}^{*tot}$ represents the rank of the total strength of node i among all nodes, and α captures the network hierarchy strength.

The Z-axis, representing the average nearest-neighbor strength [57] defined in Equations (3) and (4), captures the variation in matching strength across the staple food trade networks and thereby measures the degree of preferential connectivity among countries (or regions) within the major grain trade.

(3) $$r_{w^{1}}=\frac{H^{-1}\sum_{i}w_{i}^{1}(j_{i}k_{i})-{\left[H^{-1}\sum_{i}\frac{1}{2}w_{i}^{1}(j_{i}+k_{i})\right]}^{2}}{H^{-1}\sum_{i}\frac{1}{2}w_{i}^{1}({j_{i}}^{2}+{k_{i}}^{2})-{\left[H^{-1}\sum_{i}\frac{1}{2}w_{i}^{1}(j_{i}+k_{i})\right]}^{2}}$$

(4) $$H=\sum_{i}w_{i}^{1}$$

where $j_{i}$ and $k_{i}$ represent the degrees of the two linked nodes, the weighted assortativity coefficient $r_{w^{1}}$ accounts for the influence of supply weights, and ${\bar{K}}_{nn,i}$ represents the average nearest-neighbor strength.

**Figure 2:**
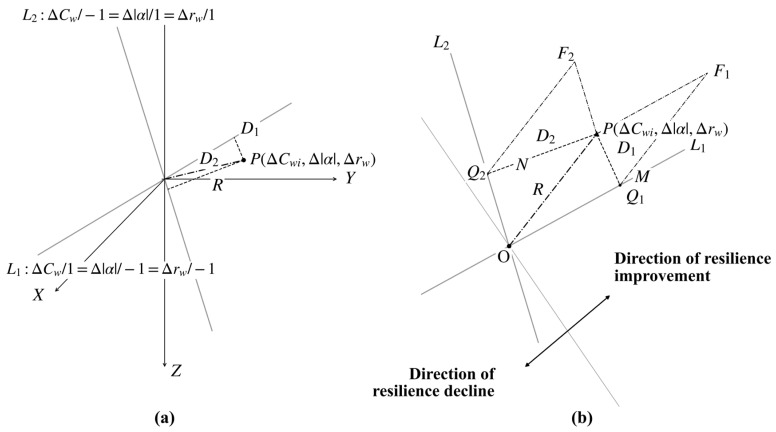
The three-dimensional framework of structural resilience. Panels (**a**) and (**b**) present the 3D demonstration and 2D mapping plots respectively.

After obtaining evolutionary data for three dimensions of grain trade networks, namely clustering (X-axis), hierarchy (Y-axis), and assortativity (Z-axis), network structural resilience is modeled as a state point $P(\Delta C_{wi},\Delta \left|\alpha \right|,\Delta r_{w^{1}})$ within three-dimensional space. The three dimensions correspond to three independent mechanisms underpinning the pressure resistance of trade networks: the density of local redundant channels, the extent of control core nodes exert over the network, and the connection preference of high-intensity nodes. The three dimensions feature low statistical correlation and functionally complementary mechanisms. Calculation of the Evolutionary Structural Resilience (ESR) (Equation (5)) index further captures the overall structural resilience evolution of the three types of trade networks from historical baselines to the present. Geometrically, line $L_{1}:\Delta C_{w}/1=\Delta \left|\alpha \right|/\left(-1\right)=\Delta r_{w}/\left(-1\right)$ represents the monotonic resilience degradation trajectory, which signals declining clustering, amplified hierarchical dominance and stronger assortativity; line $L_{2}:\Delta C_{w}/\left(-1\right)=\Delta \left|\alpha \right|/1=\Delta r_{w}/1$ denotes the monotonic resilience improvement trajectory. The ratio $D_{2}/D_{1}$, derived from the distances $D_{1}$ and $D_{2}$ between any evolutionary point P and the two reference lines, quantifies the degree to which the network evolves toward recovery or deterioration. The norm R=‖OP‖ reflects the overall evolution magnitude, and the composite metric $ESR=\pm R•\left(D_{2}/D_{1}\right)$ simultaneously characterizes the direction and intensity of resilience shifts, with the algebraic sign determined by the orientation of the cross product. Unlike conventional scalar indicators that merely detect structural degradation, geometric decomposition of the ESR index identifies both the exact dimension driving degradation and the corresponding degradation magnitude, which delivers an interpretable structural analytical framework for tracing the root causes of network vulnerability.

(5) $$ESR=\pm (R•\frac{D_{2}}{D_{1}})=\pm (\sqrt{{\left(\Delta C_{wi}\right)}^{2}+(\Delta \left|\alpha \right|{)}^{2}+(\Delta r_{w^{1}}{)}^{2}})\left(\frac{\left|\vec{OP}\times \vec{OQ_{2}}\right|}{\left|\vec{OQ_{2}}\right|}/\frac{\left|\vec{OP}\times \vec{OQ_{1}}\right|}{\left|\vec{OQ_{1}}\right|}\right)$$

where R represents the distance from the point $\left(\Delta C_{wi},\Delta \left|\alpha \right|,\Delta r_{w^{1}}\right)$ to the origin, and $D_{1}$, $D_{2}$ denote the distances from the point to lines $L_{1}$ and $L_{2}$, respectively. The cross product $\left|\vec{a}\times \vec{b}\right|$ gives the area of the parallelogram with adjacent sides $\vec{a}$ and $\vec{b}$; hence $D_{1}=\frac{\left|\vec{OP}\times \vec{OQ_{1}}\right|}{\left|\vec{OQ_{1}}\right|}$ and $D_{2}=\frac{\left|\vec{OP}\times \vec{OQ_{2}}\right|}{\left|\vec{OQ_{2}}\right|}$, as illustrated in Figure 2.

### 2.4. Construction of the Single-Layer Network Underload Cascading Failure Model

This study assesses dynamic resilience using the underload cascade failure model [51], retaining the core mechanisms and scenario settings while measuring model outputs via the cumulative integral [48]. Degree-based, betweenness-based, random, and PageRank-based attack strategies then drive underload cascade failure simulations on the wheat, maize, and rice trade networks. Node attacks fall into two categories: random and deliberate. Random attacks destroy nodes probabilistically, irrespective of topological position, and may stem from climate events, natural disasters, or geopolitical instability [66,67]. Deliberate attacks, by contrast, target critical nodes with greater network impact and may take the form of abrupt changes in trade policies of specific countries or regions, causing disruptions in food supply or demand [67,68,69]. India’s export ban, for instance, constitutes a deliberate attack intended to disrupt trade.

#### 2.4.1. Settings for Load and Capacity

Before establishing the negative attenuation and distribution rules, this study identifies three node categories determined by import and export load profiles: import type, balanced type, and export type. Import-type nodes feature import loads exceeding export loads. Balanced-type nodes feature import loads slightly less than or equal to export loads. For the two types, export loads can exist in three states: normal, attenuated, and failed; the degree of attenuation depends on the ratio of import to export loads and the attenuation state of the node. Export-type nodes, by contrast, show import loads less than export loads; export-type nodes are generally robust and assume only two states, normal and attenuated.

To meet the requirements of dynamic simulation attacks, this study defines two thresholds: the attenuation point and the failure point. The attenuation point refers to the threshold at which a node, in response to a decline in input load, actively reduces output load, reflecting the node’s buffering and coordination capabilities under shocks. The failure point refers to the threshold at which a node fails when incoming load falls below this value, whereupon outgoing load drops to zero, reflecting the bottom line and minimum tolerance of the node under shocks. Equations (6) and (7) provide the formulas for setting these thresholds:

(6) $$M_{i(dec)}=\alpha_{1}K_{i}^{in}\;0\leq \alpha_{1}\leq 1$$

(7) $$M_{i(\min)}=\alpha_{2}K_{i}^{in}\;0\leq \alpha_{2}\leq \alpha_{1}\leq 1$$

where $M_{i(dec)}$ denotes the attenuation point, $\alpha_{1}$ is the attenuation coefficient and serves as the key parameter for adjusting attenuation capacity in simulations, $M_{i(\min)}$ denotes the failure point, and $\alpha_{2}$ is the failure coefficient and serves as the key parameter for adjusting failure capacity in simulations.

Based on the node classification and threshold settings described above, this study further constructs a load attenuation function (Equation (8)) for node outputs:

(8) $$F_{i}(t)=\left\{\begin{array}{cc} 0 & K_{i}^{in}(t)\geq M_{i(dec)}\\ \frac{K_{i}^{in}(t)}{K_{i}^{in}} & M_{i(\min)}\leq K_{i}^{in}(t)\leq M_{i(dec)}\\ 1 & K_{i}^{in}(t)\leq M_{i(\min)} \end{array}\right.$$

where $F_{i}(t)$ denotes the node export load attenuation function: a value of 0 represents the normal state, 1 represents the failed state, and when $0\leq F_{i}(t)\leq 1$, the attenuated state persists for the node.

#### 2.4.2. Attenuation Function for Export Loads

For an import-type node, the output load attenuation function is governed by the import-to-output load ratio and the node’s attenuation state, as specified in Equation (9).

(9) $$\Delta K_{i}^{out}(t)=\left\{\begin{array}{cc} 0 & F_{i}(t)=0\\ K_{i}^{out}(1-\alpha_{3}F_{i}(t)) & 0<F_{i}(t)<1\\ K_{i}^{out} & F_{i}(t)=1 \end{array}\right.$$

where $\Delta K_{i}^{out}(t)$ denotes the export load reduction of node i at time t. When $F_{i}(t)=0$, node i is in the normal state and the export load reduction equals zero. When $F_{i}(t)=1$, node i is in the failed state and the export load reduction equals the total export load. When $0<F_{i}(t)<1$, node i is in the attenuated state and the export load reduction equals $K_{i}^{\mathrm{out}}(1-\alpha_{3}F_{i}(t))$, where $\alpha_{3}$ denotes the ratio of output load to input load of the node, as given in Equation (10).

(10) $$\alpha_{3}=\frac{1}{1+\ln(K_{i}^{in})-\ln(K_{i}^{out})}\;\;K_{i}^{out}\leq K_{i}^{in}$$

When node i is in the attenuated state, the magnitude of attenuation is governed by the logarithmic difference between import and export loads. Specifically, when $K_{i}^{out}\leq K_{i}^{in}$, $\alpha_{3}<1$, and a greater logarithmic difference between import and export loads leads to a smaller value, which in turn produces greater attenuation, reflecting that a country heavily dependent on imports suffers a more severe decline in export load once a cascading failure occurs.

For balanced-type nodes, the export load attenuation function. Since import and export loads of such nodes are approximately equal, the logarithmic difference between the two is around zero; taking $\alpha_{4}=1$, the function simplifies to the form given in Equation (11):

(11) $$\Delta K_{i}^{out}(t)=\left\{\begin{array}{cc} 0 & F_{i}(t)=0\\ K_{i}^{out}(1-F_{i}(t)) & 0<F_{i}(t)<1\\ K_{i}^{out} & F_{i}(t)=1 \end{array}\right.$$

For export-type nodes, the attenuation function for export loads is defined as follows. Given that export loads far exceed import loads, a shock that reduces imports to zero merely decreases exports without triggering outright failure. Consequently, the function exhibits no failure state; the large export-to-import load ratio ensures that attenuation has negligible impact on export load, and the function simplifies to the form specified in Equation (12):

(12) $$\Delta K_{i}^{out}(t)=\left\{\begin{array}{cc} 0 & F_{i}(t)=0\\ K_{i}^{out}(1-F_{i}(t)) & 0<F_{i}(t)<1 \end{array}\right.$$

#### 2.4.3. Redistribution of Export Loads

When a node falls into the attenuated or failed state, the load originally destined for downstream nodes cannot be delivered. To sustain its own functionality, the node reduces export load, and the resulting export load reduction must be shared among downstream nodes. The redistribution rule is defined as follows: if a node is in the failed state, the export load of the node drops to zero and the edge loads with all neighboring nodes also drop to zero, such that $\Delta K_{i}^{out}(t)=K_{i}^{out}$ and $\Delta K_{i}^{tot}(t)=K_{i}^{tot}$ hold. If a node is in the attenuated state, the export load reduction is distributed to downstream nodes inversely proportional to the importance of the nearest neighbors, with the detailed redistribution rule presented below:

(13) $$IM_{ij}=K_{ij}^{tot}\times EC_{j}$$

(14) $$R_{i\varepsilon_{i}}=\Delta K_{i}^{out}-\Delta K_{i\varepsilon_{1}}-\Delta K_{i\varepsilon_{2}}-\Delta K_{i\varepsilon_{3}}-\ldots -\Delta K_{i\varepsilon_{j-1}}$$

where $IM_{ij}$ represents the importance of the nearest neighbor, $K_{ij}^{tot}$ denotes the total trade volume between nodes, and $EC_{j}$ denotes the eigenvector centrality of neighbor j. Sorting by nearest-neighbor importance, $IM_{ij}$ in ascending order yields $\sigma =\varepsilon_{1},\varepsilon_{2},\varepsilon_{3},\ldots ,\varepsilon_{k_{i}}$, where $k_{i}$ is the out-degree of node i and lower-importance nodes receive allocation priority, with $R_{i\varepsilon_{i}}$ representing the remaining total amount to be allocated for node i and $\Delta K_{i\varepsilon_{j}}$ the amount allocated from node i to node j, with redistribution continuing until $R_{i\varepsilon_{i}}=0$, at which point the redistribution is complete.

Taking node $\varepsilon_{j}$ in period t as an example, the allocation amount is specified as follows:

Under the condition $\frac{k_{i}-\varepsilon_{j}}{k_{i}}>F_{i}(t)$, if the allocation amount of the node exceeds the remaining total allocation amount, specifically $K_{i\varepsilon_{j}}\times \frac{k_{i}-\varepsilon_{j}}{k_{i}}>R_{i\varepsilon_{j}}(t)$, then the node bears the entire remaining amount, such that $\Delta K_{i\varepsilon_{j}}(t)=R_{i\varepsilon_{i}}$; if $K_{i\varepsilon_{j}}\times \frac{k_{i}-\varepsilon_{j}}{k_{i}}<R_{i\varepsilon_{j}}(t)$, then the node bears the maximum allocatable amount, specifically $\Delta K_{i\varepsilon_{j}}(t)=K_{i\varepsilon_{j}}\times \frac{k_{i}-\varepsilon_{i}}{k_{i}}$. Under the condition $\frac{k_{i}-\varepsilon_{j}}{k_{i}}<F_{i}(t)$, if $K_{i\varepsilon_{j}}\times F_{i}(t)>R_{i\varepsilon_{j}}(t)$, then $\Delta K_{i\varepsilon_{j}}(t)=R_{i\varepsilon_{i}}$; otherwise $\Delta K_{i\varepsilon_{j}}(t)=K_{i\varepsilon_{j}}\times F_{i}(t)$.

#### 2.4.4. Parameter Specification and Cascade Process

Table 2 summarizes all parameters in the cascade failure model and the justification for their assigned values. We select each parameter value based on theoretical plausibility, empirical validity, and computational identifiability.

The functional decay state of node i at time t is defined by the piecewise function (Equation (8)). The node remains fully functional and no cascading failures are triggered when its current in-strength is above $\alpha_{1}\geq 0.7$ of the initial value $\left(F_{i}=0\right)$; linear functional damage occurs when the in-strength falls within the interval $\left[\alpha_{2},\alpha_{1}\right.$ $\left(F_{i}\in \left(0,1\right)\right)$, where export loads are reduced proportionally and cascading failures begin to propagate. Once the in-strength drops below $\alpha_{2}\cdot K_{in}(0)$, the node suffers complete failure $\left(F_{i}=1\right)$, and all its connected edges are removed from the network, with export-oriented nodes exempted to maintain the foundation of the global food supply.

This piecewise functional form draws upon the capacity-load tolerance model proposed by W. Tian et al. (2025) [48]. However, a heterogeneous assignment of the coefficient $\alpha_{2}$ is adopted in this work, which enables the failure threshold to reflect discrepancies in trade roles: countries with high import dependence are assigned larger values of $\alpha_{2}$.

#### 2.4.5. Resilience Evaluation Metrics

To measure the retained resilience of a network after cascading failures, this study employs the cumulative integral. Assume that the network suffers node disruptions at $t_{0}$, triggering cascading failures that continue until all nodes have failed at $t_{1}$. This study defines a resilience retention function $P^{h}(t)$ to capture the retained resilience at time t. The retained resilience $R^{h}$ then equals the definite integral of $P^{h}(t)$ from $t_{0}$ to $t_{1}$, as Equation (15) specifies:

(15) $$R^{h}=\int_{t_{0}}^{t_{1}}P^{h}(t)dt$$

Here, the retained resilience of the network is jointly measured by global efficiency, the largest connected component, and node strength, all of which are weighted indicators; to simplify computation, this study treats node disruptions as discrete events with a constant time step Δt, and different disruption sequences correspond to different permutations of the node set V for a given permutation $\sigma =\delta_{1},\delta_{2},\ldots ,\delta_{n}$, this study uses $P_{\delta_{i}}^{h}$ to denote network performance after the $\delta_{i}$ node fails, yielding a resilience retention sequence $\left(P_{\delta_{1}}^{h},P_{\delta_{2}}^{h},\ldots ,P_{\delta_{n}}^{h}\right)$; if a node fails ahead of the predetermined sequence due to cascading effects from other nodes, this study removes the node and reorders the remaining nodes.

Let $t_{\delta_{0}}$ denote the initial moment, $t_{\delta_{i}}$ the failure moment of node $\delta_{i}$, and Δt the constant interval between consecutive disruptions. Approximating the resilience retention function $P^{h}(t)$ as linear yields the following formula for the retained network resilience after sequential node failures:

(16) $$R^{h}=\sum_{i=1}^{n}\frac{\left(P_{\delta_{i-1}}^{h}+P_{\delta_{i}}^{h}\right)\times \Delta t}{2}$$

### 2.5. Construction of the Underload Cascading Failure Model for Coupled Networks

#### 2.5.1. Construction of Coupled Networks

The cross-layer substitution model offers an analytical tool for examining cascading failures across multi-layer trade networks. Buldyrev et al. (2010) proposed a percolation model for cascading failures in interdependent networks [70]; Gephart et al. (2016b) developed a model in which trade-network shocks propagate proportionally to trade flows and depend on GDP [71]; and Gao et al. (2012) identified various coupling mechanisms governing the transition from first-order to second-order phase transitions in interdependent networks [30]. To overcome the limitations of traditional single-layer networks, which cannot capture substitution effects among major staple crops, and to accurately reflect cascading feedback within the staple crop system under shocks, this study further constructs a multi-layer coupled grain trade network, denoted as $G_{2}$.

Given the substantial differences in energy content and economic value per unit mass among staple foods, directly aggregating or substituting physical trade volumes across levels can introduce dimensional errors. To address this, this study introduces a caloric equivalence conversion method. Using the FAO standard food caloric conversion coefficient $c_{k}$, this study uniformly maps the physical weight matrix W to an absolute caloric flow matrix $\tilde{W}$ and converts the weights to ${\tilde{w}}_{ij}=w_{ij}\times c_{k}$. Under the unified dimension, this study defines a multilayer coupled network as $G_{2}=\left(N,L,W_{2}\right)$, with $L=\left(W,C,R\right)$ representing the set of network layers. Unlike a single-layer network, the topological structure of a multilayer network $G_{2}$ finds its complete description in the supersymmetric block matrix $W_{2}$:

$$W_{2}=\left[\begin{array}{ccc} {\tilde{W}}^{W} & D^{W\to C} & D^{W\to R}\\ D^{C\to W} & {\tilde{W}}^{C} & D^{C\to R}\\ D^{R\to W} & D^{R\to C} & {\tilde{W}}^{R} \end{array}\right]$$

Specifically, the main diagonal blocks (${\tilde{W}}^{W},{\tilde{W}}^{C},{\tilde{W}}^{R}$) represent the intra-layer heat trade matrices within each major grain network, reflecting the actual physical flow of commodities in international markets; the off-diagonal blocks ($D^{W\to C},D^{W\to R}$) represent the inter-layer coupling matrices, where the inter-layer connections do not denote physical trade between countries, but rather the demand spillover and substitution relationships of the same node across different major grain layers. Specifically, $D^{W\to C}$ takes the form of a diagonal matrix, with the diagonal element $d_{ii}^{W\to C}$ capturing the dynamic spillover capacity—based on purchasing power and cross-elasticity—through which country $n_{i}$ converts a calorie deficit in the wheat layer (W) into new demand for the maize layer (C). Under steady-state conditions, the inter-layer coupling matrix D tends toward the zero matrix; yet when a supply shock at a node triggers a cascading failure, the off-diagonal blocks activate and become the core channels for transmitting risk and demand across layers.

#### 2.5.2. Inter-Layer Substitution Setting

Single-layer network cascade failure models typically treat unmet demand at a node directly as the system’s final loss. Yet significant substitution effects exist among different agricultural products in the staple food trade. When a severe supply shortage occurs for a particular staple crop (wheat), the crisis spreads across layers to the networks of substitute crops (maize and rice). This study allocates the shock according to trade shares and GDP-adjusted transfers to cross-layer substitutes [52], and adopts a domestic priority principle: when a shortage occurs, the affected country first reduces export volumes; if export cuts cannot fully absorb the shortage, the country seeks substitutes. To accurately quantify these cascading dynamics across multi-layered networks, this study constructs a joint allocation model that integrates economic substitution elasticities, macro-level purchasing power constraints, and network topology dependencies.

After the wheat network undergoes initial intra-layer cascade redistribution and export intervention, node i still carries an irreconcilable net deficit, denoted as $\Delta W_{i}$. This deficit translates into new demand for the maize and rice networks, denoted as $S_{i\to C}$ and $S_{i\to R}$. The following equations define the cross-layer conversion dynamics:

(17) $$S_{i\to C}=\Delta W_{i}\times \Phi \times (\frac{I_{i,C}}{I_{i,C}+I_{i,R}+\xi })\times \nu_{i}$$

(18) $$S_{i\to R}=\Delta W_{i}\times \Phi \times (\frac{I_{i,R}}{I_{i,C}+I_{i,R}+\xi })\times \nu_{i}$$

Here, $S_{i\to C},S_{i\to R}$ denote the maize and rice substitution amounts, respectively, when node i undergoes cascading failure, and $\Delta W_{i}$ represents the total substitution load of node i, namely the source that triggers cross-layer substitution.

$I_{i,C},I_{i,R}$ denote the total maize and rice imports, respectively, of node i in a normal year under the conventional trade network. If the node lacks mature trade channels in the target substitution network namely $I_{i,j}$ = 0, the absence of such channels still impedes cross-layer substitution. ξ represents a minimal smoothing constant that prevents mathematical anomalies arising from a zero denominator, thus ensuring that countries with no import history can still participate in the model calculation. Taking maize substitution as an example, ${\frac{I_{i,C}}{I_{i,C}+I_{i,R}+\xi }}_{i}$ determines the share of the total substitution load that the maize trade network should bear, thereby solving the allocation proportion problem.

$\nu_{i}$ represents the macro-level purchasing power constraint, reflecting the asymmetric vulnerability in which wealthy countries shift crises while poor countries endure hunger. The following formula expresses:

(19) $$\nu_{i}=\frac{\ln\left(GDP_{i}\right)-\ln\left(GDP_{\min}\right)}{\ln\left(GDP_{\max}\right)-\ln\left(GDP_{\min}\right)}$$

Φ denotes the global substitution willingness coefficient, capturing the system’s baseline spillover willingness, namely the residual spillover volume remaining after internal mandatory processing at a node. $\Delta W_{i}\times \Phi \times \nu_{i}$ determines the magnitude of the substitution load that the maize trade network should bear, thereby solving the allocation quantity problem.

## 3. Results

### 3.1. Basic Characteristics in the Trade Network of Staple Food

**Figure 3:**
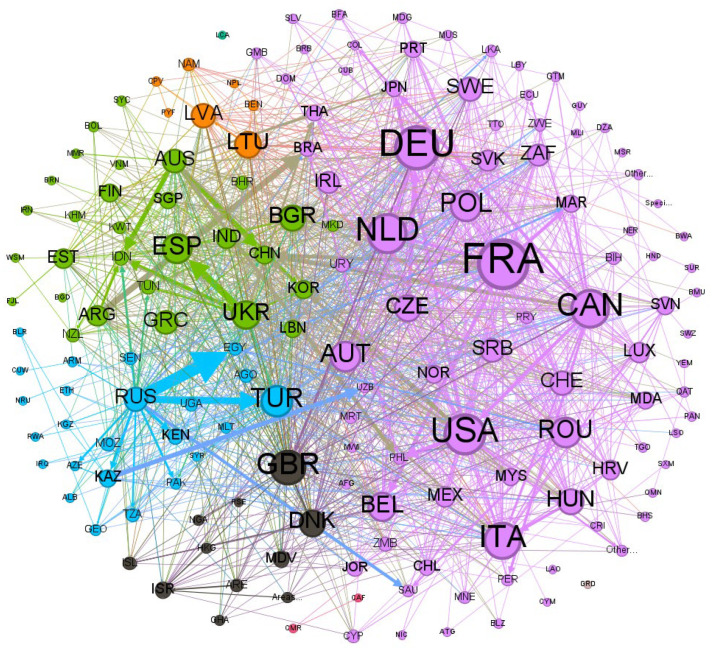
Global wheat trade network.

**Figure 4:**
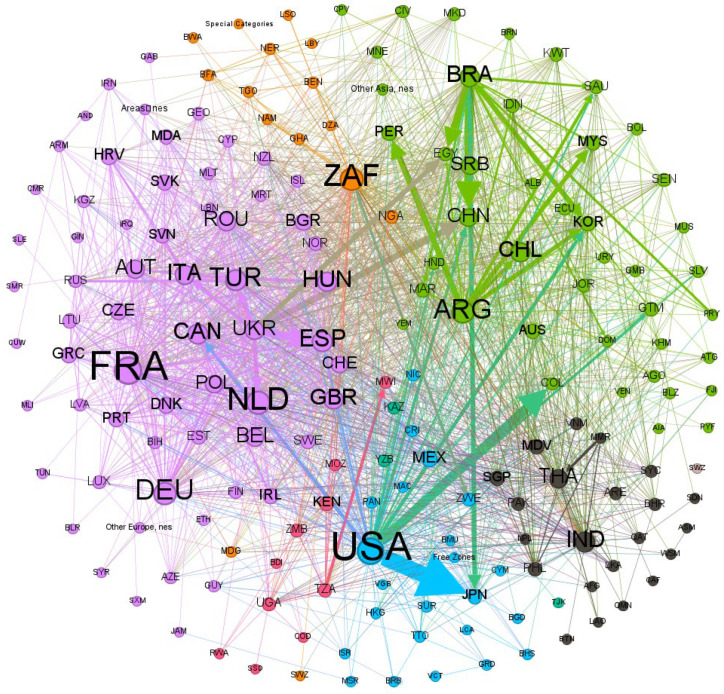
Global maize trade network.

**Figure 5:**
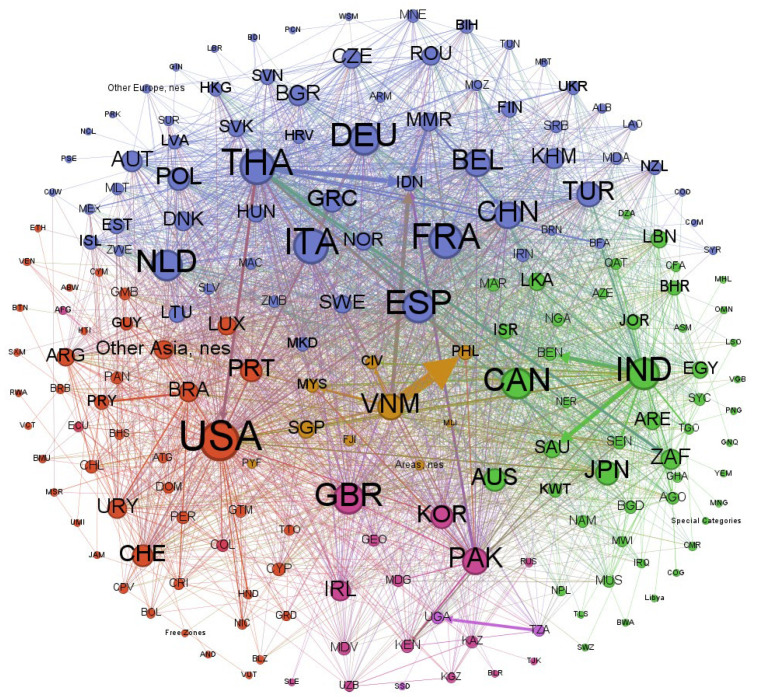
Global rice trade network.

Figure 3, Figure 4 and Figure 5, produced using Gephi (Version 0.10.1, Gephi Consortium, originally developed at the University of Technology of Compiègne, France), display the community structure of the three networks in 2024, with node colors indicating distinct modules. The wheat, maize, and rice networks contain six, seven, and seven major modules, respectively, reflecting relatively tight trade connections within each module. Wheat trade flows are heavily concentrated, with high-weight edges clustered among a few traditional exporting countries. The maize network exhibits denser trade flows, dominated by cross-continental pathways linking major suppliers to key demand regions. In contrast, the rice network displays a more diversified and decentralized structure, characterized by multiple regional hubs and elevated overall network density.

Table 3, Table 4 and Table 5 document the topological evolution of the wheat, maize, and rice trade networks from 2015 to 2024. The rice network has consistently maintained substantially larger numbers of nodes and edges than the other two, with the node count around 200, edge count exceeding 3300, and density fluctuating between 0.089 and 0.093, displaying the highest connectivity and underscoring the entrenched stability of rice as an Asian staple. This high-density connectivity provides substantial structural redundancy, effectively suppressing cascade failures triggered by local supply chain disruptions and thereby enhancing network resilience [25]. Between 2015 and 2023, maize network edges rose from 2404 to 2615, peaking at 2743 in 2021, while density increased from 0.067 to 0.074, signaling sustained expansion and deepening trade links driven by growing global feed and industrial demand. Over the same period, the wheat network—the smallest in scale—experienced node fluctuation from 181 to 175 and edge fluctuation from 1777 to 1740, yet density edged up from 0.055 to 0.057, pointing to a consolidation of core trade links. This trend of shrinking scale alongside tightening connections indicates that the wheat trade network is evolving toward a more centralized core. Although this consolidation strengthens the risk resistance capacity within the core group, from a network topology perspective, such heightened concentration also severely restricts the alternative pathways through which peripheral countries can access resources, thereby exacerbating global food distribution inequality and systemic vulnerability [71].

**Figure 6:**
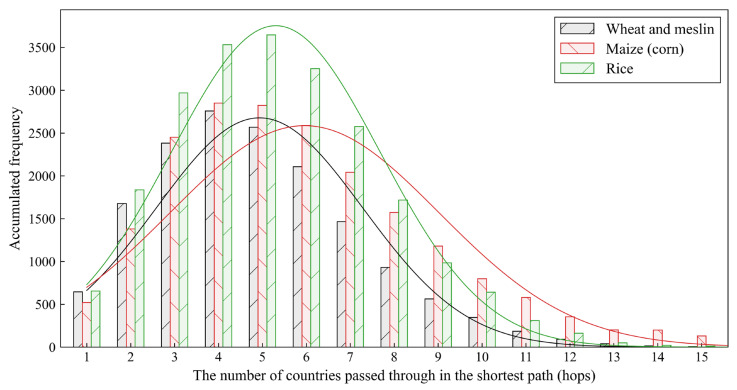
Characteristics of the shortest path length distribution.

Figure 6 depicts the distribution of weighted shortest path lengths across the wheat, maize, and rice trade networks. All three networks exhibit a normal distribution of path lengths, ranging from 1 to 15, where a length of 1 indicates direct trade and a length of 2 or more signals potential indirect channels. Although the wheat and rice networks contain comparatively more direct routes than maize, the share of direct routes remains low overall, revealing intrinsically limited resource transfer efficiency. Notably, the rice network records the highest frequency of shortest paths traversing 3–7 countries, implying the greatest abundance of alternative routes and adaptive resilience under supply disruptions. However, lengthy indirect trade chains also significantly increase the potential risk of volatility transmission. From the perspective of global cascading failures, an extended path length implies a stretching of the shock propagation chain. Once a transit hub country experiences disruption due to geopolitical conflict or logistical bottlenecks, the supply crisis can readily permeate and amplify through multiple layers of indirect network nodes, thereby intensifying the implicit food security risks faced by underdeveloped peripheral importing countries [72].

### 3.2. Evolution Analysis of Static Structural Resilience

#### 3.2.1. Single-Dimensional Analysis

**Figure 7:**
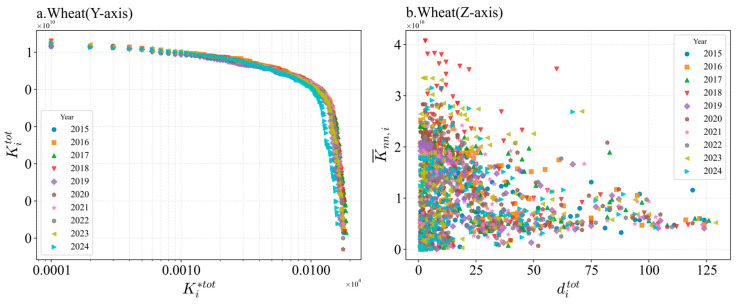
Strength and strength correlation distributions of the wheat trade network.

**Figure 8:**
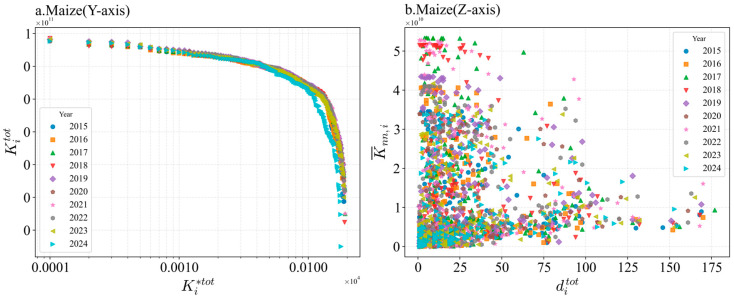
Strength and strength correlation distributions of the maize trade network.

**Figure 9:**
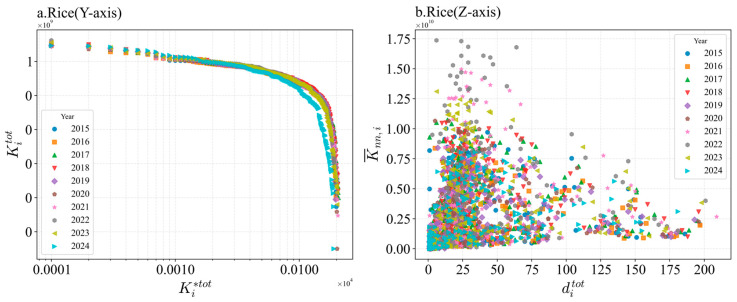
Strength and strength correlation distributions of the rice trade network.

Figure 7, Figure 8 and Figure 9 present the hierarchical distributions of node strength and the nearest-neighbor strength correlation distributions for the wheat, maize, and rice trade networks from 2015 to 2024, based on Equations (2) and (3). In terms of hierarchical structure, the node strength of all three networks approximately follows a power-law distribution, characterized by a gradual decline at early ranks and a sharp decline at intermediate and later ranks as rank increases, indicating that global staple food trade networks exhibit pronounced scale-free properties. This coexisting scale-free and disassortative topological structure endows global staple food trade networks with a typical robust-yet-fragile character [73]. Although the network exhibits strong resilience to localized random market fluctuations, the highly asymmetric dependence of peripheral countries on core exporting hubs renders it highly susceptible to the rapid cascading diffusion of policy or physical shocks targeted at core nodes, such as export bans or geopolitical blockades, toward underdeveloped peripheral nations, thereby exacerbating the unequal vulnerability of global food security [74].

Regarding the nearest-neighbor strength correlations, all three networks display disassortative mixing characteristics. This disassortativity is more pronounced in the wheat and maize networks, where the heavy reliance of importing countries in Africa and the Middle East on a few major exporters elevates the nearest-neighbor strength of low-strength nodes. In contrast, the disassortative pattern in the rice network is more entrenched; though it generally exhibits robust resistance to random disruptions, its dependence on core nodes renders it more susceptible to targeted attacks.

**Figure 10:**
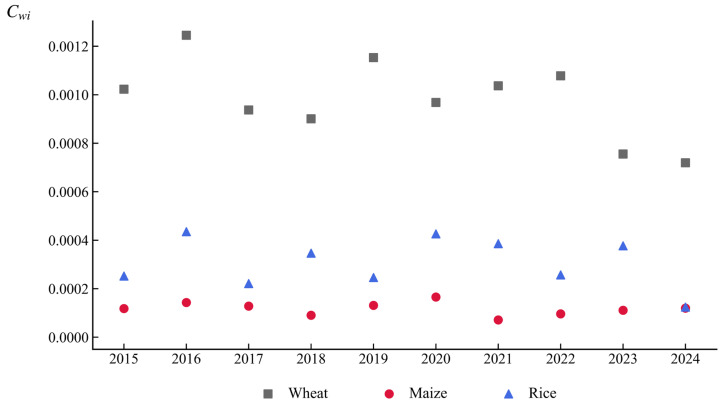
The clustering of the staple food trade networks.

Figure 10 depicts the changes in clustering coefficients across the three networks. The wheat network exhibits the highest clustering coefficient, possessing abundant alternative routes and thus strong redundancy and buffering capacity, whereas the rice network displays a relatively low coefficient and the maize network remains at a persistently low level. This indicates that the maize network depends on a limited number of backbone links and offers restricted buffering capacity under localized disruptions. Regarding evolutionary trends, the wheat and rice networks exhibit a gradual decline, reflecting an erosion of cohesion within the trade networks. The volatile decline of the clustering coefficient, particularly the recent downward trend observed in the wheat and rice networks, fundamentally reveals a trend toward de-communalization and fragmentation in global staple food supply chains, driven by geopolitical conflicts and trade protectionism. Lower clustering implies that neighboring countries find it difficult to form mutually supportive trade clusters, rendering the network highly dependent on individual cross-regional trade corridors. This topological evolution weakens intra-regional self-organizing resilience; once a systemic disruption occurs at core global supply hubs, importing countries that lack the protection of localized clusters become directly exposed to the risk of supply chain rupture [43].

#### 3.2.2. Three-Dimensional Evolution Analysis

**Figure 11:**
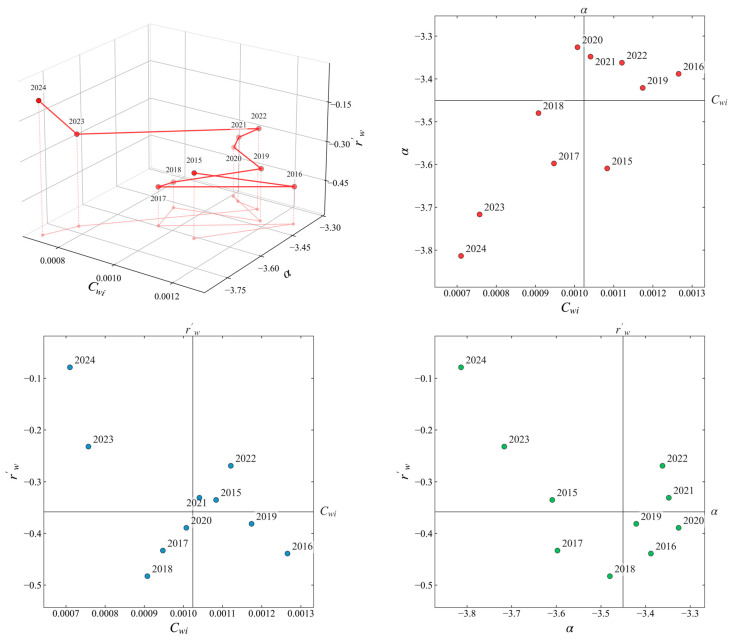
Three-dimensional structural resilience evolution and two-dimensional projection of the wheat trade network.

**Figure 12:**
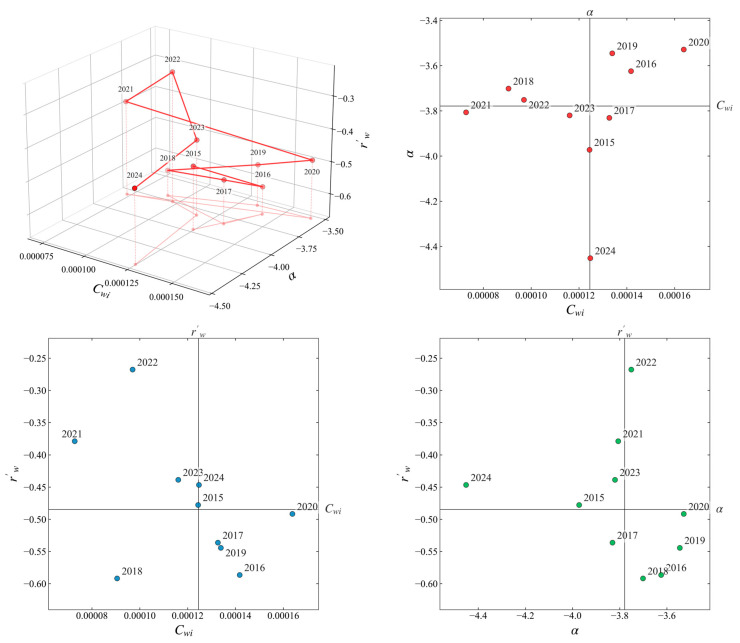
Three-dimensional structural resilience evolution and two-dimensional projection of the maize trade network.

**Figure 13:**
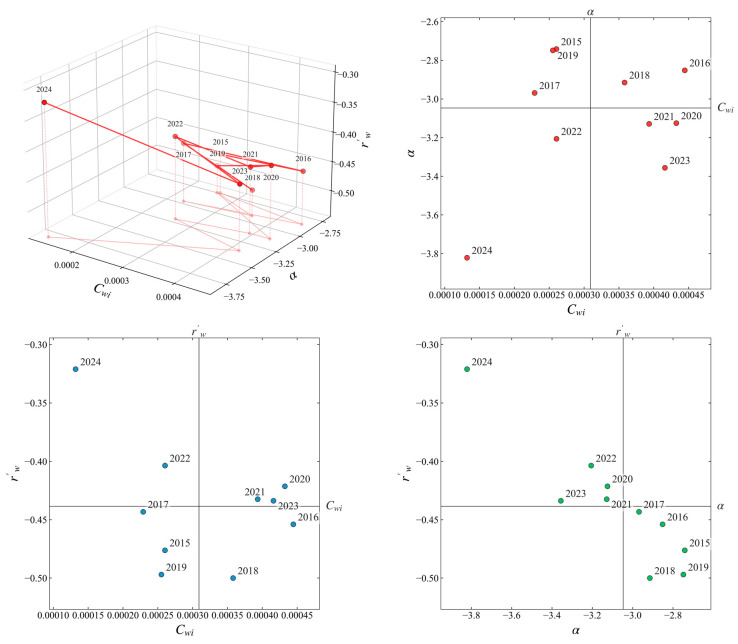
Three-dimensional structural resilience evolution and two-dimensional projection of the rice trade network.

This section synthesizes the hierarchical, matching, and clustering properties to visualize the three-dimensional evolutionary trajectories and their two-dimensional projections using Python (Version 3.14.2, Python Software Foundation, Wilmington, DE, USA)-based tools. Figure 11, Figure 12 and Figure 13 display the three-dimensional structural resilience evolution and two-dimensional projection of the staple food trade network.

Figure 11, Figure 12 and Figure 13 demonstrate that the structures of the three grain trade networks underwent multi-phased shifts in response to external shocks between 2016 and 2024, ultimately restabilizing through the reallocation of trade flows. Pandemics, geopolitical conflicts, and trade frictions primarily reshape trade flows and partner compositions by disrupting key supplier countries, resulting in synchronous short-term fluctuation of clustering, hierarchy, and matching. Over the long term, all three networks exhibit a general trend toward channel expansion and source diversification, yet they experience temporary concentration in years of severe shocks.

Figure 11 illustrates the four key phases of the wheat trade network. In 2017, reduced production in Australia curtailed exports, compelling importers to broaden their partner base and causing simultaneous declines in both centralization and hierarchy. In the early stages of the pandemic, from 2019 to 2020, food security concerns concentrated trade flows more heavily toward core importing nations, elevating the hierarchical structure. This concentration effect reflects that, when facing sudden public health crises, countries tend to consolidate existing core trading channels to reduce transaction costs and manage supply uncertainty [75]. In 2022, the Russia–Ukraine conflict and disruptions to Black Sea shipping prompted importers to shift toward alternative suppliers such as Australia, Canada, and India, substantially reconfiguring the network. From 2023 to 2024, the network continued to evolve toward a more decentralized pattern. This forced diversification adjustment is fundamentally a self-organizing resilience mechanism of the network driven by geopolitical crises. Although it increases the frictional costs of reconfiguring trade channels in the short term, the dispersal of trade linkages breaks the previous rigid dependency and steers the wheat network toward a decentralized dynamic equilibrium.

As depicted in Figure 12, the maize trade network underwent structural reconfiguration in 2018, when U.S.–China trade friction led China to significantly reduce imports from the United States and redirect purchases to Brazil and Argentina. This drastic adjustment, triggered by strategic competition among major powers, vividly illustrates how unilateral external policy interventions can instantaneously reshape the topological linkages and resource flows of global staple food trade networks [25]. In 2019–2020, the pandemic, combined with reduced production in Argentina, further concentrated trade flows and increased network vulnerability; the network subsequently recovered and has trended toward decentralization in recent years. Under the dual pressures of persistent climate change and an unstable multilateral trade regime, this trend toward decentralization essentially represents a proactive defensive strategy adopted by importing countries to mitigate the risks associated with highly concentrated single-market dependence, aiming to dilute the disruption threat posed by specific export nodes through the construction of a multilateral supply chain matrix.

Figure 13 shows the evolution of the rice trade network. Between 2017 and 2019, India leveraged its price advantage to capture market share from Thailand and Vietnam, weakening the formerly concentrated structure. From 2023 to 2024, India’s ban on non-Basmati rice exports intensified supply-side constraints, concentrating trade flows on a few alternative suppliers, abruptly altering the network structure, and triggering a market reconfiguration. India’s unilateral export restrictions, as the world’s largest rice exporter, directly severed multiple previously efficient trade pathways, plunging highly import-dependent countries into structural supply bottlenecks [76]. Since the global rice trade is inherently a rigid structure characterized by high local dependency, the supply vacuum created by export restrictions is extremely difficult for other actors to fill in the short term. This not only triggers cascading supply chain disruptions but also, more fundamentally, exposes the systemic vulnerability of global staple food networks when a key hub imposes a strategic embargo.

Table 6 shows that the ESR of the trade networks for the three major staple grains diverged significantly from 2016 to 2024. The wheat ESR fluctuates frequently between positive and negative values, exhibiting the greatest volatility and reflecting a resilience subjected to repeated disturbances and recovery. In contrast, the maize ESR turned negative in certain phases and continued to weaken, with a gradually widening negative margin, indicating deepening structural imbalances. The rice network’s ESR, meanwhile, remained negative over the long term, with only brief positive spells in a few years, reflecting sustained pressure on the resilience of the rice network within a relatively rigid supply-demand structure. This evolutionary characteristic captures, at the level of complex physical systems, the fundamental differences in intrinsic tolerance among the three major staple grain networks when subjected to sustained external perturbations. According to the resilience theory of dynamical network systems, the capacity to maintain macroscopic stability depends on the speed of adaptive topological reorganization [49]. The wheat network demonstrates a strong dynamic corrective capacity through frequent pathway substitution; the maize network, with its persistent negative deviation, signals a deepening structural imbalance that warrants global concern; and the rice network remains in a marginal critical state of resilience, urgently requiring multilateral coordination to inject structural flexibility.

### 3.3. Evolution Analysis of Dynamic Resilience

#### 3.3.1. Cascading Failure Pathways Under Core Node Failure

**Figure 14:**
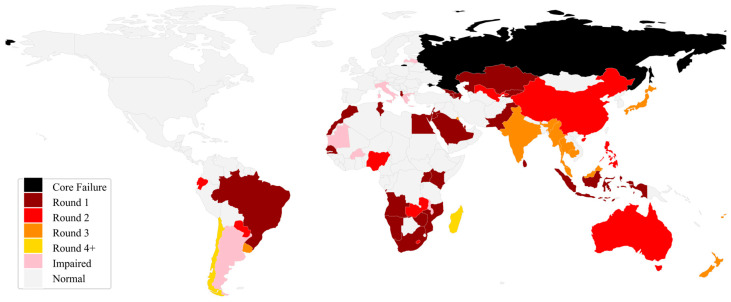
Underload cascade failure propagation map of the 2024 wheat trade network.

Based on a simulation of network-level cascading failures in the 2024 staple grain trade, Figure 14 demonstrates that the wheat network exhibits a sequential geographical propagation pattern, with Russia serving as the primary failure source. The failure propagates via a first round of direct disruptions affecting Turkey, Egypt, North Africa, and Brazil; a second round affecting China, Japan, and sub-Saharan Africa and finally a deeper-level cascade affecting India and Pakistan.

**Figure 15:**
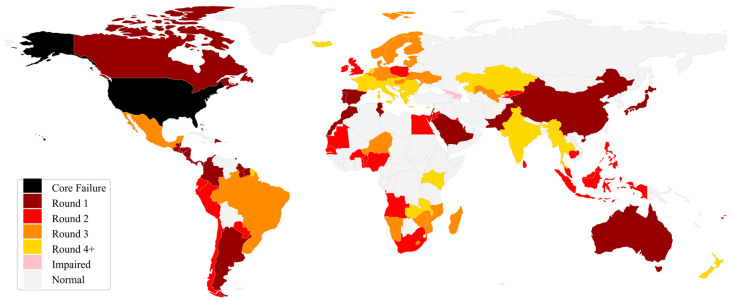
Underload cascade failure propagation map of the 2024 maize trade network.

With the United States at its core, the maize market precipitated simultaneous market disruptions across multiple regions, including Canada, Mexico, South America, Africa, the Middle East, and East Asia, with a scope substantially exceeding that of other commodities (Figure 15).

**Figure 16:**
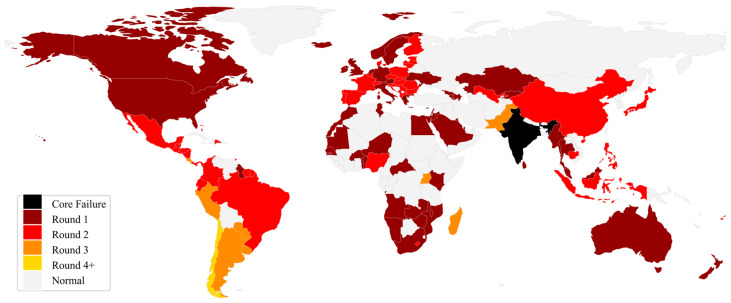
Underload cascade failure propagation map of the 2024 rice trade network.

The rice network centers on India, echoing the country’s recent export restrictions. Vietnam, Thailand, Myanmar, and regions in the Middle East and Africa constitute the primary shock zones, whereas China occupies the secondary zone (Figure 16). Although geographically concentrated, the disruption threatens South and Southeast Asian regions home to over two billion rice-dependent people, signaling that the human risk far outweighs the physical footprint of the crisis.

#### 3.3.2. Cascading Failure Results Under Single-Layer Networks

To investigate the dynamic evolution of resilience in the global staple food trade network, this study employs four node removal strategies, namely random perturbation, out-degree strength, betweenness centrality, and PageRank centrality, to assess the resilience of the 2024 global staple food trade network under individual node disruption scenarios, as well as resilience trends from 2015 to 2024.

**Figure 17:**
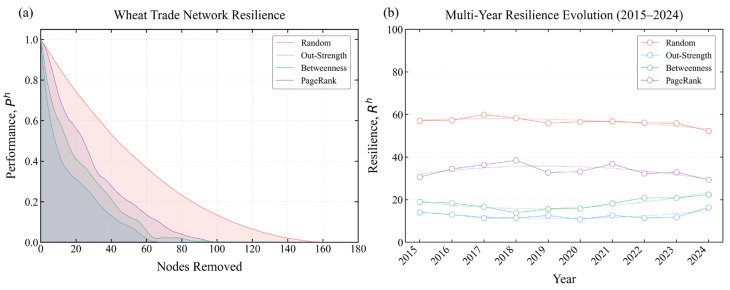
Network performance retention and evolution of the wheat trade network.Panel (**a**) shows the resilience decline rate of cascading failures in 2024, and Panel (**b**) presents the resilience change curve from 2015 to 2024.

**Figure 18:**
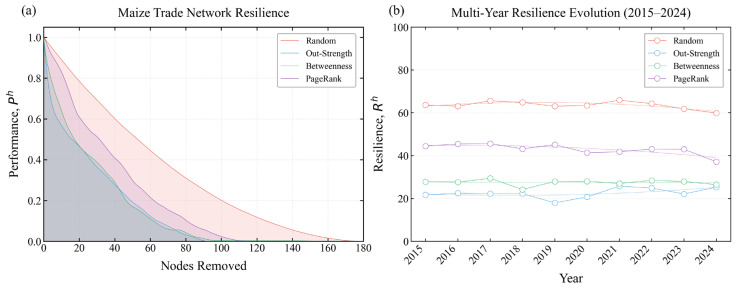
Network performance retention and evolution of the maize trade network.Panel (**a**) shows the resilience decline rate of cascading failures in 2024, and Panel (**b**) presents the resilience change curve from 2015 to 2024.

**Figure 19:**
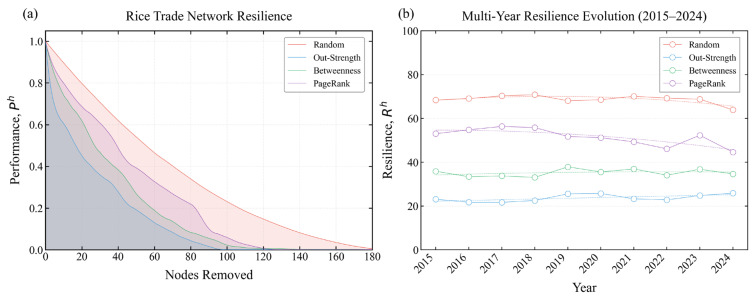
Network performance retention and evolution of the rice trade network.Panel (**a**) shows the resilience decline rate of cascading failures in 2024, and Panel (**b**) presents the resilience change curve from 2015 to 2024.

Figure 17, Figure 18 and Figure 19 demonstrate that, across all three networks, resilience retention rates under random perturbation are consistently higher than those under targeted attacks; out-degree strength-based and betweenness centrality-based attacks can rapidly induce network collapse, underscoring the dominant position of a few core exporting nations in global food trade networks. This topological asymmetry, marked by high tolerance to random fluctuations and extreme sensitivity to the removal of critical nodes, clearly confirms a defining characteristic of complex networks under systemic risk [49]. When hub nodes with high out-degree or betweenness are disrupted, the network’s global connectivity collapses precipitously, abruptly severing the links between peripheral importing countries and the global supply chain. Although the multi-year resilience curves remained generally stable, they declined discernibly under major shocks—as exemplified by the downward trend observed across all three networks during the COVID-19 pandemic of 2019–2020 [77].

As shown in Figure 17, the removal of as few as 60–70 core nodes in the wheat network drives network performance to near zero; the PageRank-based attack exerts intermediate destructive power, and the 2022 Russia–Ukraine conflict precipitated a sharp decline in wheat network resilience. The out-degree strength-based and betweenness centrality-based attack curves for the maize network overlap closely, with complete collapse following the removal of approximately 80–90 nodes (Figure 18). The shaded area between the random perturbation curve and the targeted attack curves is larger than that observed in the wheat network, indicating greater susceptibility to targeted attacks. In 2019, maize network resilience under out-degree strength-based attacks reached a distinct nadir, attributable to the U.S–China trade friction. The rice network exhibits the highest resilience to random perturbations, owing to substantial network scale and high connection density (Figure 17), yet the network is the most susceptible to targeted attacks, with performance falling below 0.2 upon the removal of as few as 40–50 nodes. The network also displays a highly concentrated out-degree distribution, and the random resilience index of the network declined markedly in 2024, driven by India’s export restrictions.

#### 3.3.3. Simulated Attack Comparison Based on Real-World Scenarios

To further enhance the credibility of the cascade failure model, this study uses India’s wheat export ban in May 2022 as a natural experiment to evaluate the predictive capacity of the improved model. We take the 2021 wheat trade network, based on HS 1001 and comprising 179 nodes and 1802 edges, as the baseline, apply differentiated export shocks, run the cascade model, and compare the output with actual trade data from 2022. The ten countries most dependent on Indian wheat are selected as the validation sample.

To adapt the cascade failure model to the export ban verification scenario, we introduce three modifications. First, the attack mode is changed from node removal to differentiated edge-weight reduction, reflecting the tendency of an exporting country to prioritize its core trading partners under a ban. The higher an importing country’s share of India’s exports, the greater its edge-weight retention rate, which ranges from 15% to 65%. Second, the cascade triggering condition is shifted from node deactivation to an import shortfall rate: each country compares its current imports with the baseline, and the shortfall drives a proportional reduction in its own exports via a transmission coefficient β. Third, a node collapse threshold is introduced, defined as a shortfall rate of 80% or above, to simulate the functional exit of severely disrupted countries from the market. The improved model preserves the iterative convergence framework and the shortfall transmission logic of the original model, while offering a more realistic representation of the attack.

**Figure 20:**
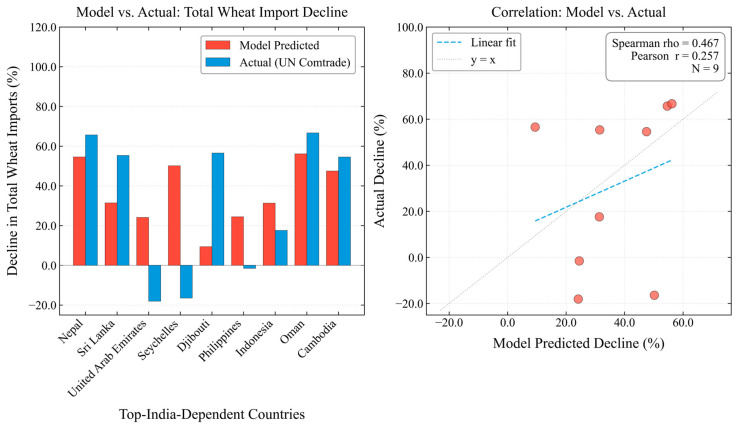
Comparison of simulated and real-world scenarios.

Verification results shown in Figure 20 indicate that the model achieves a Spearman rank correlation coefficient of 0.467 between the predicted ranking of total import change rates and actual values, effectively distinguishing high-vulnerability countries from low-vulnerability ones. At the structural level, the node strength of the post-shock network correlates with that of the real 2022 network at 0.948, with *p* below 0.001, and the cascade does not trigger spurious network collapse. The number of edges decreases by only 0.9%, while the average path length and clustering coefficient remain consistent with the baseline. Three factors account for the limited predictive precision. First, Indian wheat exports account for merely 3.2% of global trade edges, and the dominant driver of network changes in 2022 was the Russia–Ukraine war rather than the Indian ban; any single-country shock model cannot fully explain the total variation. Second, the model does not incorporate alternative supplier mechanisms and therefore cannot capture the resilient behavior of countries such as the United Arab Emirates, which increased imports by shifting to sources in Russia and Australia. Third, the sample covers only nine countries highly dependent on India, and this small sample size limits statistical significance, with *p* equal to 0.205. Despite deviations in point predictions, the model’s core value lies in its conservatism and structural stability: it produces no spurious amplification effects and preserves the network topology, thereby providing a reliable foundation for subsequent multi-country joint shock simulations.

#### 3.3.4. Cascading Failure Results Under Coupled Networks

**Figure 21:**
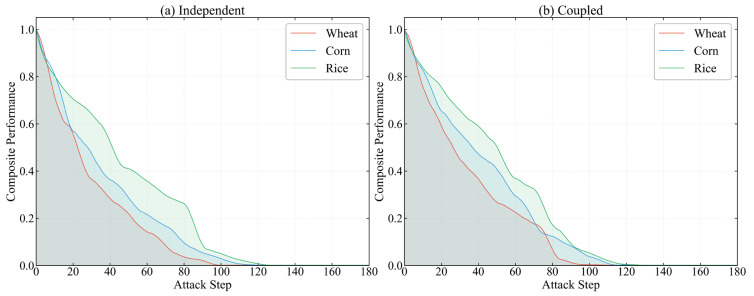
Single-Layer versus Coupled Network Resilience under Individual Attack Experiments. Panel (**a**) shows the resilience decline rate under single-layer network attacks, and Panel (**b**) shows the resilience decline rate under coupled network attacks.

**Figure 22:**
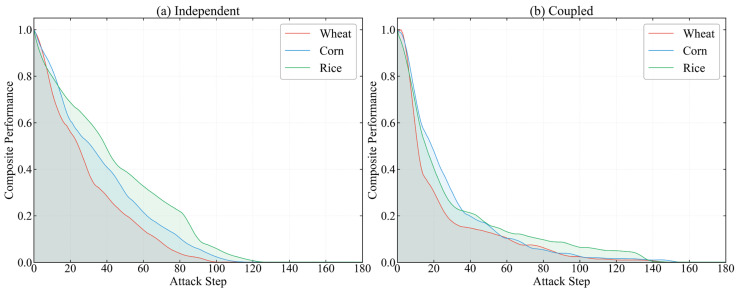
Single-Layer versus Coupled Network Resilience under Simultaneous Attack Experiments. Panel (**a**) shows the resilience decline rate under single-layer network attacks, and Panel (**b**) shows the resilience decline rate under coupled network attacks.

This study incorporates crop substitution effects and cross-layer network substitution into the coupled network model, and conducts single-attack and simultaneous-attack experiments on three staple grain trade networks. Figure 21 and Figure 22 present the results. The single-attack experiments simulate a global shortage of a specific grain, attacking only a single trade network, while the other two remain operational, as exemplified by the Russia–Ukraine conflict and India’s rice export ban. The simultaneous-attack experiments simulate concurrent shortages across multiple grain types, attacking all three networks simultaneously, as exemplified by global extreme weather events and strong El Niño phenomena.

As shown in Figure 21, under a single-attack scenario, the resilience of all three crop networks dropped by more than 90% within approximately 80 steps. After introducing a cross-layer substitution mechanism, Figure 21b shows that the attacked layer can draw resources from other normal layers to compensate for the gap. The resilience curves of the three crops rise markedly within the first 40 steps, and the system collapse time is delayed by approximately 20–30 steps, verifying the buffering effect of the multi-layer coupled substitution mechanism against single-point shock under simultaneous-attack experiments (Figure 22), by contrast, the performance of the three networks degraded far more rapidly than in the single-layer scenario; removing as few as 20 nodes reduced the retention rates of all three networks to below 0.2. These findings indicate that the crop substitution effect nullified the structural buffering advantage inherent in single-layer networks, and the coupling instead became a transmission channel for rapid cross-network failure propagation [39].

**Figure 23:**
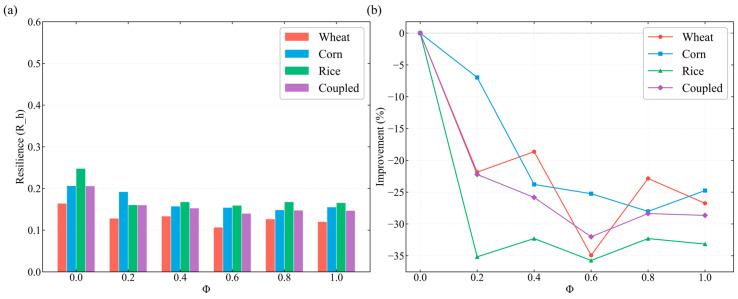
Comparison of resilience improvement at different substitution willingness levels. Panel (**a**) shows the resilience of different networks under varying substitution willingness, and Panel (**b**) presents the trend of resilience for different networks as substitution willingness changes.

Figure 23 presents the cascading failure results of coupled networks under simultaneous attack experiments for varying levels of substitution willingness. Figure 23a demonstrates that the rice network exhibits the highest resilience under zero substitution willingness, yet the disparities among crops narrow markedly as substitution willingness increases. Figure 23b reveals the resilience retention rate relative to the no-substitution-willingness scenario. All four networks experience a precipitous decline in resilience when substitution willingness reaches 0.2, indicating that even a modest degree of cross-crop substitution can exert a substantial adverse effect on network structure. The rice network proves the most susceptible to substitution willingness; when substitution willingness reaches 0.8, the relative resilience loss attains approximately 37%, reflecting that the structure of the rice network is particularly vulnerable to the reconfiguration of cross-crop trade flows.

## 4. Conclusions and Discussion

This paper constructs directed weighted trade networks for global wheat, maize, and rice based on UN Comtrade from 2015 to 2024 and employs a three-dimensional structural resilience framework and underload cascading failure models to systematically evaluate the robustness of these trade networks. Simulation results reveal:

(1) The wheat network has decentralized its resources and diversified its partnerships, whereas the maize network continues to scale up. Meanwhile, the rice network preserves a highly diversified and tightly connected structure; collectively, all three exhibit scale-free and disassortative topologies. A three-dimensional structural resilience assessment reveals that direct trade accounts for only a minor share across these networks, restricting their transmission efficiency. However, the rice network provides the most abundant alternative pathways, securing the highest resilience against supply chain disruptions.
(2) Cascading failure pathways differ sharply across the three crops. Wheat supply shocks propagate sequentially along traditional supply chains. Conversely, maize’s deep integration into global feed and biofuel sectors triggers widespread, simultaneous disruptions worldwide, with North America acting as the epicenter of this global interdependence. Meanwhile, rice failures remain geographically concentrated due to export restrictions, yet their societal footprint is immense, impacting billions of people.
(3) Simulations of single-tier cascading failures  reveal that disrupting just a few key exporters triggers systemic chain reactions, yielding losses that far exceed those from random shocks. Each crop displays a distinct vulnerability profile: rice boasts the highest resilience to random disturbances yet depends acutely on pivotal suppliers; maize succumbs easily to targeted attacks, particularly disruptions arising from Sino–US strategic competition; and wheat, while relatively balanced, remains susceptible to global systemic crises like the COVID-19 pandemic [23].
(4) In a coupled network incorporating crop substitution effects, substitutes from other grains can offset a shortage in a particular grain trade network under normal conditions, thereby enhancing the resilience of the affected network to external shocks. Yet under a global external shock, when shortages occur simultaneously across multiple grain crops, cross-layer substitution creates pathways for supply gaps across different crop networks. Coupled with the networks’ scale-free and heterogeneous characteristics, such substitution transfers collapse pressure from core exporting countries to other cross-layer importing countries, so that the coupled network not only fails to buffer risks but triggers an even more severe overall collapse [70].

The key innovations of this study include:

(1) This study introduces a three-dimensional dynamic framework to assess global staple food trade networks. By tracking network restructuring over the past decade—amid overlapping geopolitical conflicts, pandemics, and unilateral trade bans—we quantify how different grain networks exhibit vulnerability, recovery, and rebalancing dynamics across these compounding crises.
(2) Applying an under-capacity cascade failure model to resilience analysis of wheat, maize, and rice trade networks overcomes the limitations of conventional static studies by simulating cascading failures under four attack strategies, thereby identifying cross-border failure transmission pathways and offering new methods for the early warning of disruption risks.
(3) By incorporating crop-substitution willingness, this newly developed multi-level coupled model advances network resilience research from isolated, single-system investigations to an integrated, multi-dimensional framework.

Although this study yielded valuable findings, several limitations suggest avenues for future work. The substitution mechanism assumes direct caloric-based transfers under globally uniform parameters, yet actual substitution is constrained by cultural preferences, dietary habits, infrastructure, and price elasticities. Future research should incorporate region-specific substitution elasticities and distinguish short-term emergency responses from long-term structural adjustments. The model’s deterministic node behavior also cannot capture strategic government or enterprise responses, such as diplomatic negotiations, reserve releases, and temporary trade agreements, or speculative behaviors; agent-based or game-theoretic approaches could enhance realism. Furthermore, the annual, national-scale framework overlooks intra-annual seasonality and subnational heterogeneity, while fixed inter-layer substitution matrices fail to reflect the dynamic evolution of substitution channels during crises. Adopting finer spatiotemporal resolutions and adaptive network frameworks with endogenous coupling strengths would improve the model’s scientific rigor.

Global staple food trade governance urgently requires a tiered, interconnected security mechanism. First, establishing differentiated strategic reserves among the top five core exporters of wheat, maize, and rice, and implementing export stabilization agreements, will mitigate the ripple effects caused by the failure of key hubs. Second, establishing a cross-crop substitution threshold early-warning system, activating cross-layer allocation only upon a localized shortage in a single crop, and automatically switching to a regional rationing priority mode when two or more crop categories face simultaneous shortages, prevents the risk of reverse transmission through substitution channels. Third, promoting the institutionalized sharing of rice substitution channels and guiding countries dependent on wheat and maize imports to proactively diversify their trade partners enhances the overall network’s resilience against systemic shocks. Furthermore, for export-dependent major economies, establishing an export safeguard mechanism anchored in out-strength can prevent abrupt export controls from triggering global cascading failures, thereby sustaining stable output from core hubs. For import-dependent countries, it is essential to actively hedge against the strong dependency risks arising from disassortativity and low clustering. Through import diversification and regional multilateral mutual assistance alliances, these countries can shorten their shortest import paths and achieve localized self-healing when core nodes are subjected to targeted attacks.

## Figures and Tables

**Table 1:** Static Characteristic Indicators of Complex Networks.

Assessment Dimensions	Evaluation Metrics	Key References
Redundancy	density	Che et al. (2022) [44]; Miao et al. (2024) [40]
	Average degree	Kim et al. (2015) [45]
	Number of node edges	Rings et al. (2022) [46]; Xu and Xu (2024) [47]
Connectivity	Global efficiency	W. Tian et al. (2025) [48]; Yu et al. (2024) [49]
	Average path length	Xing and Yuan (2025) [50]
	Largest connected component	Huang et al. (2025) [51]; Keefe et al. (2024) [52]
Clustering	Clustering coefficient	Fagiolo (2007) [53]; Li et al. (2022) [54];
Hierarchicality	Gini coefficient	Park and Newman (2003) [55]
Matchability	Pearson’s correlation coefficient	Ash and Newth (2007) [56]; Pigorsch and Sabek (2022) [57]
Core dimensions	Coreness, k-shell index	Wan et al. (2021) [58]; Wu et al. (2024) [59]
Local connectivity	Average neighbor strength	Pigorsch and Sabek (2022) [57]; Jafari et al. (2023) [60]

**Table 2:** Parameter descriptions and justifications.

Parameter	Value	Description	Justification
$\alpha_{1}$	0.7	Node functionality degradation threshold	Threshold set as 70% of initial in-strength, following the tolerance parameter of ~0.3; results remain directionally robust under 5–10% perturbations.
$\alpha_{2}$	(0.0–0.7)	Lower-bound coefficient for complete node failure	Graded assignment by node type: strong importer, importer, balanced, exporter, strong exporter
$\alpha_{3}$	Import: $1/(1+\ln(K^{in})-\ln(K^{out}))$ Balance: 0.5 Export: 0.1	Export load attenuation ratio	Export-oriented nodes retain a stronger spillover capacity, while for import-oriented nodes, logarithmic scaling captures the property that their spillover elasticity declines as the trade surplus decreases.
$\theta_{i}$	$K_{i}^{in}/K_{i}^{out}$	Node trade orientation skewness	Used to classify nodes into five categories: strong importer (θ≥2), importer (1≤θ<2), balanced (0.5≤θ<1), exporter (0.2≤θ<0.5), and strong exporter (θ<0.2) [51].
N_MC	100	Number of Monte Carlo repetitions	Balances statistical stability, with the standard error below 1%, against computational feasibility.
Attack steps	180	Maximum number of attack rounds per simulation	Set slightly above the largest connected component size to ensure complete decay to collapse across all scenarios.

**Table 3:** Fundamental parameters of the wheat trade network.

Year	Nodes	Edges	Average Degree	Density
2015	181	1777	9.818	0.055
2016	179	1781	9.95	0.056
2017	187	1788	9.561	0.051
2018	185	1692	9.146	0.05
2019	174	1657	9.523	0.055
2020	177	1700	9.605	0.055
2021	180	1802	10.011	0.056
2022	178	1703	9.567	0.054
2023	175	1740	9.943	0.057
2024	163	1621	9.945	0.061

**Table 4:** Fundamental parameters of the maize trade network.

Year	Nodes	Edges	Average Degree	Density
2015	190	2404	12.653	0.067
2016	188	2422	12.883	0.069
2017	193	2492	12.912	0.067
2018	192	2468	12.854	0.067
2019	188	2550	13.564	0.073
2020	189	2542	13.45	0.072
2021	194	2743	14.139	0.073
2022	192	2699	14.057	0.074
2023	188	2615	13.91	0.074
2024	180	2412	13.4	0.075

**Table 5:** Fundamental parameters of the rice trade network.

Year	Nodes	Edges	Average Degree	Density
2015	199	3499	17.583	0.089
2016	201	3602	17.92	0.09
2017	207	3706	17.903	0.087
2018	206	3905	18.155	0.089
2019	197	3675	18.655	0.095
2020	203	3691	18.182	0.09
2021	205	3892	18.985	0.093
2022	203	3828	18.857	0.093
2023	202	3779	18.708	0.093
2024	190	3323	17.489	0.093

**Table 6:** Temporal dynamics of structural resilience in staple food trade networks.

Network	Index	2016	2017	2018	2019	2020	2021	2022	2023	2024
Wheat	ESR	0.244	−0.209	0.128	0.117	0.095	0.062	0.064	−0.356	0.182
Maize	ESR	0.365	−0.213	0.141	0.163	0.055	−0.299	0.124	−0.184	−0.632
Rice	ESR	−0.112	−0.117	−0.078	0.166	−0.384	−0.012	−0.082	−0.153	−0.479

## Data Availability

The original contributions presented in this study are included in the article. Further inquiries can be directed to the corresponding authors.
